# Patterns of Leaf Biochemical and Structural Properties of Cerrado Life Forms: Implications for Remote Sensing

**DOI:** 10.1371/journal.pone.0117659

**Published:** 2015-02-18

**Authors:** Aaron Ball, Arturo Sanchez-Azofeifa, Carlos Portillo-Quintero, Benoit Rivard, Saulo Castro-Contreras, Geraldo Fernandes

**Affiliations:** 1 Earth and Atmospheric Sciences Department, University of Alberta, Edmonton, Alberta, Canada, T6G 2E3; 2 Ecologia Evolutiva de Herbívoros Tropicais/DBG, CP 486, ICB/Universidade Federal de Minas Gerais, 30.161–970, Belo Horizonte, MG, Brazil; University of A Coruña, SPAIN

## Abstract

**Aim:**

The general goal of this study is to investigate and analyze patterns of ecophysiological leaf traits and spectral response among life forms (trees, shrubs and lianas) in the Cerrado ecosystem. In this study, we first tested whether life forms are discriminated through leaf level functional traits. We then explored the correlation between leaf-level plant functional traits and spectral reflectance.

**Location:**

Serra do Cipo National Park, Minas Gerais, Brazil.

**Methods:**

Six ecophysiological leaf traits were selected to best characterize differences between life forms in the woody plant community of the Cerrado. Results were compared to spectral vegetation indices to determine if plant groups provide means to separate leaf spectral responses.

**Results:**

Values obtained from leaf traits were similar to results reported from other tropical dry sites. Trees and shrubs significantly differed from lianas in terms of the percentage of leaf water content and Specific Leaf Area. Spectral indices were insufficient to capture the differences of these key traits between groups, though indices were still adequately correlated to overall trait variation.

**Conclusion:**

The importance of life forms as biochemical and structurally distinctive groups is a significant finding for future remote sensing studies of vegetation, especially in arid and semi-arid environments. The traits we found as indicative of these groups (SLA and water content) are good candidates for spectral characterization. Future studies need to use the full wavelength (400 nm–2500 nm) in order to capture the potential response of these traits. The ecological linkage to water balance and life strategies encourages these traits as starting points for modeling plant communities using hyperspectral remote sensing.

## Introduction

**Fig 1:**
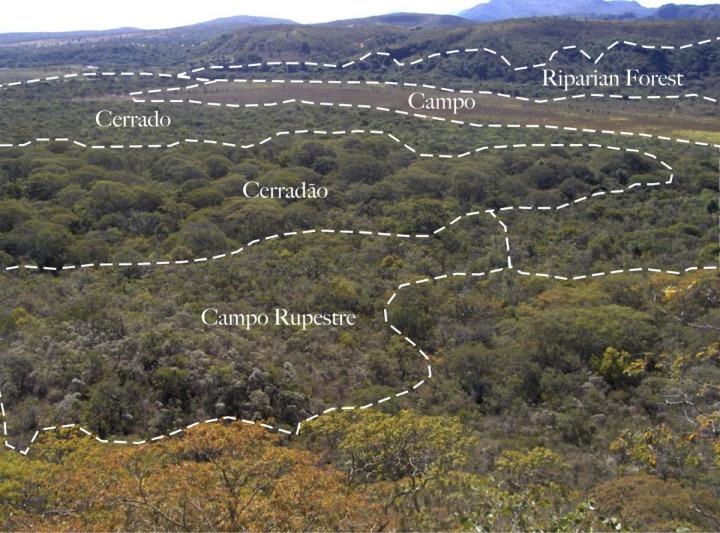
Physiognomic types of the Cerrado *sensulato* ecosystem in the study area of Serra do Cipò, NP. The entire visible landscape together is Cerrado *sensulato*. The vegetation subtypes are texturally distinguishable and outlined. Campo rupestre is on the rocky hill in the foreground. The canopy trees beyond that are Cerradão, followed by the shorter sparse canopy of Cerrado *sensu stricto* and a quick transition into the grassland Campos beyond that.

The Brazilian Cerrado is the largest and most diverse tropical savannah. This ecosystem is generally identified as a biodiversity hotspot containing close to 44% of endemic plant species [1]. Cerrado *sensu stricto* is its archetypical physiognomy: An understory of shrubs and grass, covered by patchy to moderate canopy closure of contorted trees and lianas up to 14 m in height [2]. The ecosystem is characterized by a gradient of vegetation density; ranging from shrubby savannah to tropical dry forest [3] with up to 1,500 woody plant species [4], and 12,000 vascular plant species. The gradient of vegetation physiognomy varies from a completely closed with restricted understory (Cerradão) to grassland with shrubs (campo sujo) or without (campo limpo). In soils with low nutrient levels (above 1000 masl), the Cerrado is represented by a unique physiognomy called rupestrian field or campo rupestre (Fig. 1) [5]. Although different terms can be given to each vegetation subtype, the term Cerrado usually refers to the region as a whole.

In spite of the Cerrado’s high conservation value, continued anthropogenic disturbance and deforestation of the Cerrado has transformed the ecosystem to such extent that is now being considered as critically endangered [6–7]. Deforestation rates within the Cerrado are estimated to be higher than those in the Amazon [4], thus placing it amongst the tropical biomes under the most severe threat in the recent decades.

One of the main challenges for the scientific understanding and conservation of the Brazilian Cerrado is the scarcity of available information on the geographical extent and spatial distribution of its different plant associations or functional types. The ranges of values across functional traits, or the number and characteristics of functional types present in an ecosystem strongly influence its short-term fluxes of matter and energy [8]. Identifying the spatial and temporal patterns of its functional diversity (types, life forms or life strategies) is essential to understand it’s functioning and it’s ability to cope with climatic stresses. Furthermore, the identification of trait-spectra relationships is fundamental to designing future remote sensing missions that can provide more comprehensive information of life forms rather than the typical forest vs. non-forest classifications. This is even more relevant for life forms such as lianas, since they have been identified as increasing as a result of human disturbance and climate change. Trait-spectra derived monitoring mechanisms will provide essential information to design effective conservation strategies and program at the local and regional level, where ecosystem’s adaptation to climate change will to play an important role on their design.

Recent developments in remote sensing of vegetation, at different spatial and temporal scales, have allotted the possibility of using remotely sensed spectral data to detect the location and spatial distribution of specific plant groups at the landscape level [9–11]. These studies are founded on the basis of the spectral properties of vegetation, its woody and photosynthetic components, which has been extensively characterized in the last few decades [12–14]. In general, spectral signatures of plants are largely similar in the visible, near- and short- wave infrared region of the spectrum, in the 400 nm to 2500 nm range [12], with their primary defining features controlled by photosynthetic pigments, water absorption features, leaf structures, lignin, and proteins [14–15]. Significant work has been done in spectral and leaf trait separation of different vegetation functional types or life forms in semi-arid ecosystems, such as tropical dry forests (e.g. [15–20]). In studies by [19] and [20], the authors contrasted leaf traits and spectral signatures of lianas and tree communities from a tropical dry forest and a tropical rainforest in Panama, Central America. These studies reported significant biochemical and spectral differences in pigment concentration (chlorophyll and carotenoids) and leaf structure between trees and lianas in tropical dry forests, but not in rainforest sites. The study suggests that water stress can induce the observed differences between lianas and trees. Lianas that cope with the dry season are characterized by having deep roots and efficient vascular systems to maintain higher leaf water content, and higher chlorophyll and carotenoid concentrations than trees. Their findings also suggest that higher spectral reflectance, higher transmittance, and lower absorbance in lianas could be acting as physiological adaptations to reduce heat load, leaf-to-air vapor pressure differences, and the potential for water stress in drier and hotter environments. A study by [21] evaluated Photochemical Reflectance Index (PRI) values from leaf hyperspectral data collected from early, intermediate and late successional stages of tropical dry forests in the Pacific Coast of Mexico. Early-stage plots were characterized by a greater presence of shrubs and grasses. The intermediate and late successional stage plots were dominated by trees. In this study, significant differences were found in terms of leaf traits and species composition between early/intermediate stages and late successional stages. Consequently, earlier stages exhibited lower PRI values, which was indicative of greater investment in photoprotective pigments (xanthophylls and carotenes) relative to chlorophyll by species in earlier successional stages. Overall, results from these studies suggest that plant adaptation strategies to semi-arid and arid climates exacerbate leaf biochemical and structural differences between life forms, with a subsequent effect on their optical properties.

Studies assessing the spectral separability of life forms are rare for the Cerrado ecosystem. [22] analyzed the spectral response of Cerrado physiognomies using a hyperspectral space-borne imaging spectrometer (EO-1 Hyperion). The study found significant differences in the Red/Near Infrared and Shortwave Infrared regions of the spectrum between shrub and tree dominated cerrado physiognomies. [23] also found spectral separability between cerrado physiognomies when contrasting spectral indices versus basal area measurements.

Although these studies have started to address the subject, the leaf biochemical properties of life forms in the Cerrado and their hyperspectral signature remain to be understood. In this context and as a first approach to fill the knowledge gaps in remote sensing of Cerrado’s functional diversity, the general goal of this study is to investigate and analyze patterns in ecophysiological leaf traits and spectral response among life forms of the Cerrado ecosystem. We first tested whether life forms are discriminated by leaf level functional traits and then, we explored the correlation between leaf-level plant functional traits and spectral reflectance indices.

## Materials and Methods

**Fig 2:**
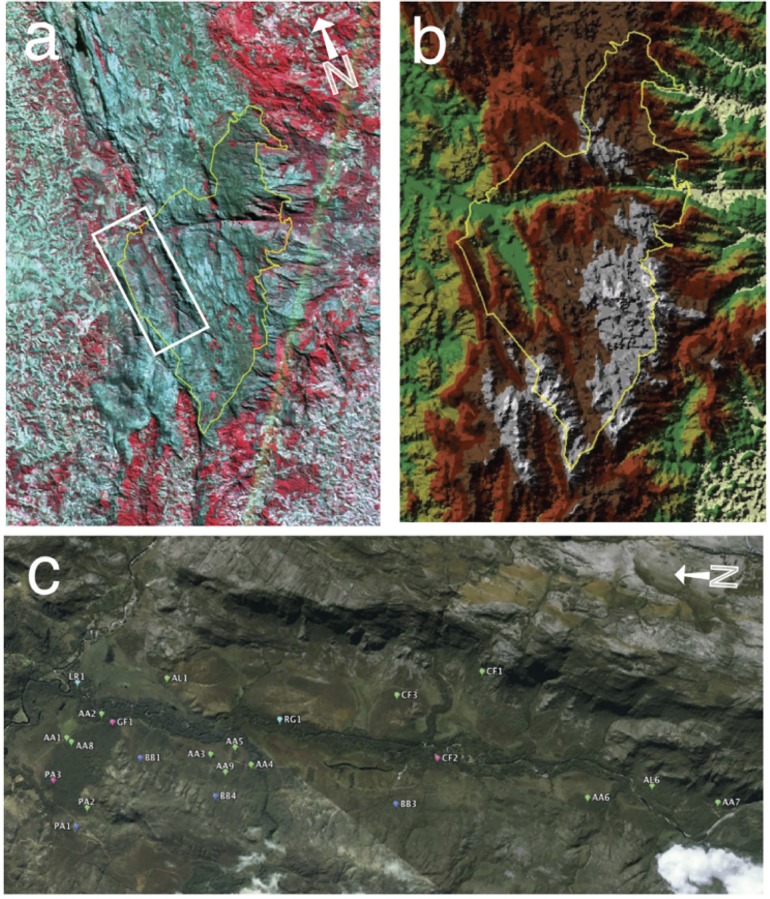
Location of the study site. a) Landsat TM false colour 30m (bands 1,3,7). Park outline is in yellow, study area in white. The national park is adjacent to the Espinhaço mountain range. Red areas are vegetated, to the NE primarily rupestrian fields, to the W Cerrado and Agriculture. b) DEM of the park and study area. c) Google Earth (2003) imagery of the study area with site locations.

This study was conducted within the Cerrado vegetation of Serra do Cipó National Park in Minas Gerais, Brazil (S 19.36°, W 43.60°). All plants used in this study were sampled during two dry seasons during the months of June to July of 2007 and June to August of 2008. Plants were sampled at pseudorandom locations (n = 21) identified with the help of a Geographic Information System (GIS) (ArcGIS, Esri, CA, USA). Using a GIS, polygons were generated over a geodatabase depicting vegetation physiognomies for the region (Figs. 1 and 2). Sampling locations were limited to the Cerrado *sensu stricto*, Cerradão, and Rupestrian Grassland physiognomies. Twelve Cerrado *sensu stricto* sites were preferentially selected relative to other physiognomies. At each sampling location in the field, specimens of woody plants corresponding to three life forms [trees, shrubs and lianas] were collected. Samples were taken within a 30-meter diameter patch of contiguous physiognomy. Even though grasses were an abundant component of the Cerrado vegetation, they were not sampled because they were all nearly completely senescent or dormant during the dry season.

Plant individuals were selected for sampling based upon those most abundantly and most frequently encountered species across the landscape. This method was chosen based on the theoretical assumptions by [24], which suggest that sampling up to 80% of the total community of all plant species in an area is sufficient for assessing the community’s functional diversity.

Plants that were more abundant across all sites and/or comprised substantial percentages of the canopy were preferentially sampled. Logistics and time constraints for collecting equal samples in all trait measures for every individual proved to be an impossible task. However, complete datasets with measurements for all traits were prioritized accounting for the most prevalent species.

For each plant species, we collected herbarium samples of branches, leaves, and inflorescences (whenever possible). Digital photos for each sample were taken to support species identification. Personnel at the Universidade Federal de Minas Gerais (UFMG, Belo Horizonte, Minas Gerais, Brazil) provided expert identification of plant species.

Between 10 and 30 sunlit, healthy leaves with no signs of herbivory or disease, were taken from each specimen and put into bags to prevent desiccation and its effects on spectral response [15]. Shade leaves were excluded because these are not representative of canopy reflectance [25]. Following collection, spectral measurements in the Visible and Near-Infrared spectrum (VNIR) were taken for every leaf sample following the protocols described by [26]. In addition, an estimate of UV reflectance was measured to predict polyphenol content in each leaf. Leaves were also subjected to different physical and chemical measures for photosynthetic pigment concentrations, specific leaf area (SLA), and water content. Some trait analyses were destructive and prevented simultaneous measurement of all traits (e.g. pigment coring preventing water content). A detailed description of the protocols for spectral measures and pigment extraction is described below.

### Spectral Measures

Leaf spectra were obtained using a portable spectrometer (UNISPEC SC, PP-Systems Analytical Spectral Devices, MA, U.S.A.) equipped with a leaf clip of 2.0 mm aperture. All spectra were converted to bidirectional reflectance by dividing the spectral data collected by the radiance measured from a barium-sulphate standard and the internal halogen light source. Three UNISPEC readings per leaf were collected. Each measurement was taken in the areolar space proximal to the medial axis while avoiding innervations. Spectral measurements were made at the tip, at the middle, and at the base of the leaf, and averaged to account for within-leaf variation. On highly serial compound leaves, such as those from the Fabaceae family, readings were done on the small leaf units near the end, the middle, and the base of the series. Spectral measurements between 450 nm and 1050 nm were taken. Spectral bands outside of this range were discarded as these bands are affected by the instrument’s noise. A spectral profile for each individual leaf was created by averaging all spectral measurements.

Using the hyperspectral data collected, we compared trait data aggregated by life form to common indices used to detect chlorophyll and water concentrations. We performed comparisons to the following spectral indices: Water Band Index (WBI = R_960_/R_900_); modified NDVI at 705 nm (mND_705_ = ((R_750_-R_705_)/(R_750_+R_705_–2R_445_)); Simple Ratio (SR = R_704_/R_774_); Gitelson-Merzylak A (G-M_A_ = R_750_/R_700_); and Gitelson-Merzylak B (G-M_B_ = R_750_/R_550_) [32–33]. Although all indices were meant to approximate the detection of chlorophyll using wavelength center values of LANDSAT bands used to calculate NDVI, the mND_705_ was developed specifically for the UNISPEC instrument [32]. It is important to stress that given the limitation of the UNISPEC after 900 nm, ecophysiological trait data and its relationships to spectral reflectance could not be tested beyond this wavelength.

Polyphenol concentrations were assessed through a UV-excitable chlorophyll absorbance index that was measured using a Dualex-FL 3.3 (Force-A, 91405 Orsay Cedex, France). UV absorbance was determined using a ration of chlorophyll fluorescence between the red and UV wavelengths. Dualex measures can be used as a linear estimate of the leaf polyphenol content, which is an indicator of the degree of photoprotection to UV radiation [26]. Dualex measurements were taken for both adaxial and abaxial sides of the leaves. The Index for polyphenol content was then calculated based on the following equation established by [27–29]:

$$Ephen=\frac{\left(adaxial\;+\;abaxial\right)}{\varepsilon }$$

Where **ε** = molar extinction coefficient equal to 20 micro mol^-1^ cm^2^ at 375 nm. Expressed in equivalents of quercetin (aglycone). The term Ephen corresponds to the measured polyphenol content index.

### Pigment Extraction

Photosynthetic pigments were extracted and subsequent in vivo spectro-photometric absorption analyses were made following methods described by [30–31]. Core sections of the leaves were extracted from healthy tissue close to the center of each leaf, avoiding major veins or innervations. These were kept airtight at -20° C in a freezer in the field and then moved for longer-term storage (up to 1 week) in at -70° C in a freezer prior to pigment extraction. Due to the high sclerophylly of many leaves and to increase solvent emulsion, sections were thoroughly ground before they were immersed in 10 mL 80% acetone and distilled water. Solutions were kept cold and dark for 24 hours while undergoing extraction and then were filtered, centrifuged (5000 RPMs for 8 minutes), and spectroscopically assessed for absorbance using a CIRRUS 80MB Spectro-Photometer (Femto, São Paulo, Brazil) at 470 nm, 645 nm and 663 nm wavelengths. Measurements were calibrated with a reference absorbance spectrum of 80% acetone. Calculations of chlorophyll a, chlorophyll b and carotenoid concentrations (mg/g) follow those presented in [30–31]. To correct for carotenoid absorptive effects in chlorophyll feature regions, the following equations were used:

CHLa=12.21*A663-2.81*A646

CHLb=20.13*A646-5.03663

$$CAR\;=\;\frac{1000\;*\;A470-3.27\;*\;CHLa-104\;*\;CHLb}{198}$$

Where CHL_a_ = Concentration (μg ml^-1^) of Chlorophyll a in 80% acetone solution (units), CHL_b_ = Concentration (μg ml^-1^) of Chlorophyll b in 80% acetone solution, CAR = concentration (μg ml^-1^) of carotenoids in 80% acetone solution standardized for the absorbance of chlorophylls at 470 nm. A = the wavelength (nm) of absorbance measurement (e.g. A_663_ represents the absorbance of the CHL_a_ at 663 nm).

Pigment concentrations were then converted to molar units (μmol m^-2^) using the respective pigment molar masses and the consistent circular area from the leaf cores using the following equation:

$$\mu mol\;=\;\frac{(Pigment)*(Molar\;Mass)*P}{100}*\;\frac{9}{\pi }$$

Where (Pigment) is the pigment concentration, and (Molar Mass) is specific to the chlorophyll a, b, or carotenoid.

### Specific Leaf Area

Specific leaf area (SLA) was assessed by scanning fresh leaves in a desktop home scanner and calculating the area digitally, using Adobe Photoshop CS3. Leaves were weighed when wet, and then oven-dried at 60° C until there was no change of weight loss from the previous test. The resultant difference of the measured water content, and the remaining dry weight was used to calculate specific leaf area in tandem with the calculated leaf area mentioned above. Water content was measured as the ratio between wet and dry leaf weights. The resulting difference in grams corresponded to the dry leaf weight.

### Statistical Analyses

ANOVAs were used to test the variance of ecophysiological trait values between trees, shrubs and liana life forms. For each ecophysiological trait, one-way ANOVAs were used to investigate the differences in the variation of trait values between life forms. The correlation between the traits was also investigated.

## Results

A total of 336 plant specimens were sampled from 21 sites. Plants from the five Cerradão sites and four rupestrian grassland sites contained families that also existed in the Cerrado *sensu stricto* sites. More than two thirds of the plant species in secondary ecotypes were encountered in the Cerrado sites as well. Of the life form groups, lianas had fewer numbers of species than shrubs or trees.

**Fig 3:**
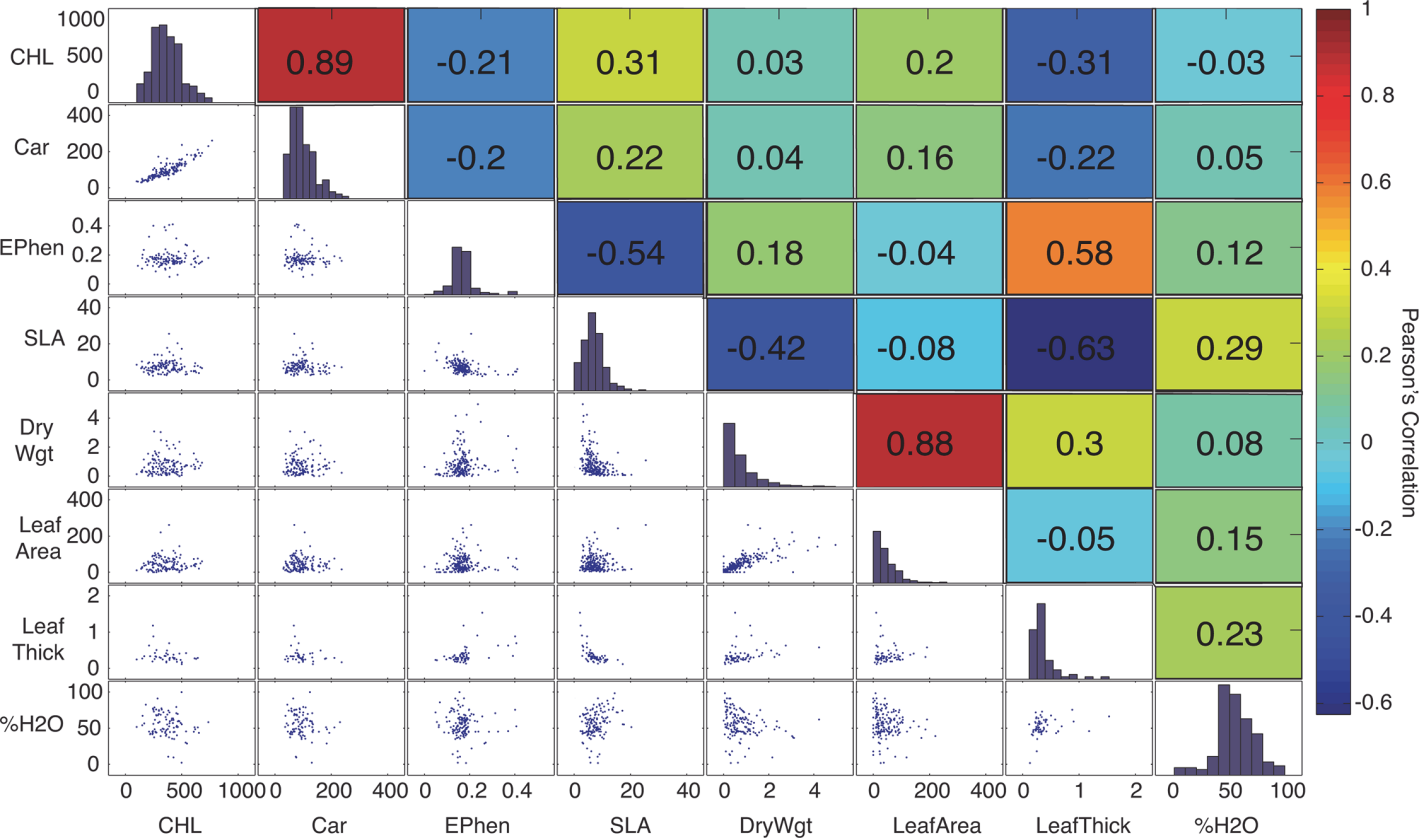
Leaf trait distributions, relationships and correlation (Pearson’s R) across all individuals. CHL = Total Chlorophylls A + B (mol), Car = Carotenoids (mol), EPhen = Polyphenol content, SLA = Specific Leaf Area (m2 kg-1), LeafArea = Leaf surface area (cm2), LeafThick = Leaf thickness (mm),%H2O = Percentage Leaf Water Conent

**Fig 4:**
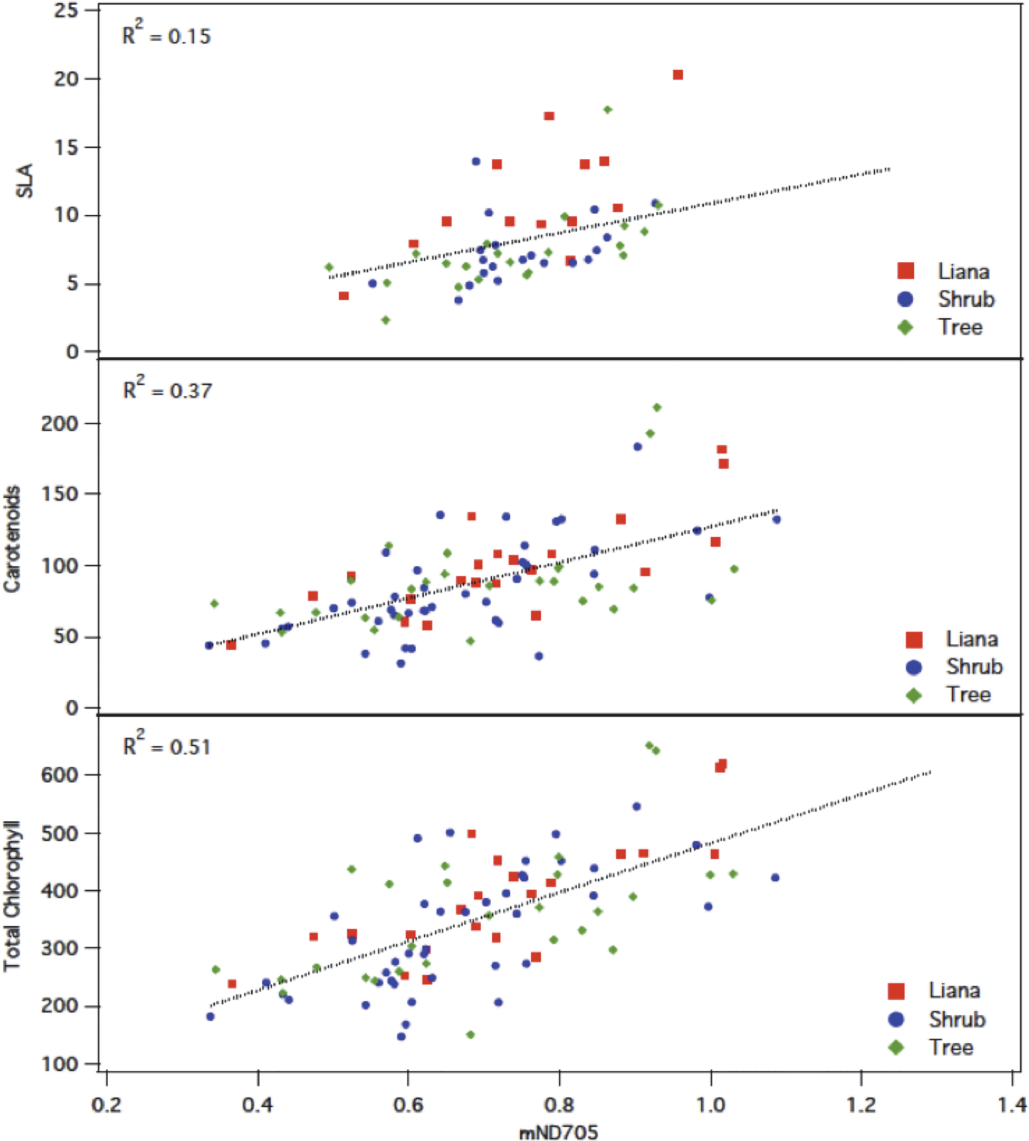
Relationships between and modified NDVI at 705nm (mND705) and Specific Leaf Area (SLA), Carotenoids and Total Chlorophyll concentration for Lianas, Trees and Shrubs.

Results from the analyzed traits by life forms are shown in Table 1. The relationship between leaf trait values is shown in Fig. 3. As expected, leaf level pigment ratios (total chlorophyll to carotenoid) were strongly correlated (r^2^ = 0.76, *p* < 0.0001) as well as leaf area and dry weight (r^2^ = 0.63, *p* < 0.0001). Polyphenol content (Ephen) was positively correlated with leaf thickness (r^2^ = 0.23, *p* < 0.0001) but negatively correlated with SLA (r^2^ = 0.33, *p* < 0.0001). Specific leaf area was not significantly related with concentrations of chlorophylls (r^2^ < 0.001, *p* = 0.77) or carotenoids (r^2^ = 0.002, *p* = 0.64). Fig. 4 shows the relationship observed between mND_705_ correlations between the different traits and mND_705_ show similar behavior as other studies [20] but no separation between functional groups is observed over a wide range of values for trees, shrubs and lianas.

**Table 1:** Summary statistics for woody plant species of the Cerrado.

	**Liana**		**Shrub**		**Tree**	
	**Average**	**Standard Deviation**	**Average**	**Standard Deviation**	**Average**	**Standard Deviation**
Chlorophyll A + B	387.1	104.4	351.3	126.6	387.6	147.4
Carotenoids	96.33	35.35	97.81	41.75	99.14	50.39
Ephen	0.149	0.04	0.169	0.057	0.174	0.059
SLA	9.031	4.901	6.101	3.456	6.744	3.816
LT mm	0.2335	0.0564	0.3927	0.2428	0.3601	0.2333
LeafArea	39.539	29.786	42.231	39.884	50.89	49.18
% Water Content	65.02	14.6	55.29	17.19	51.19	16.75
DryWgt	0.4598	0.3408	0.7547	0.8899	0.8479	0.7164

CHL = Total Chlorophylls A + B (mol), Car = Carotenoids (mol), EPhen = Polyphenol content,%H2O = Percentage. Leaf Water Content, SLA = Specific Leaf Area (m2 kg-1), LeafThick = Leaf thickness (mm), DryWgt = Dry weight (grams)

**Fig 5:**
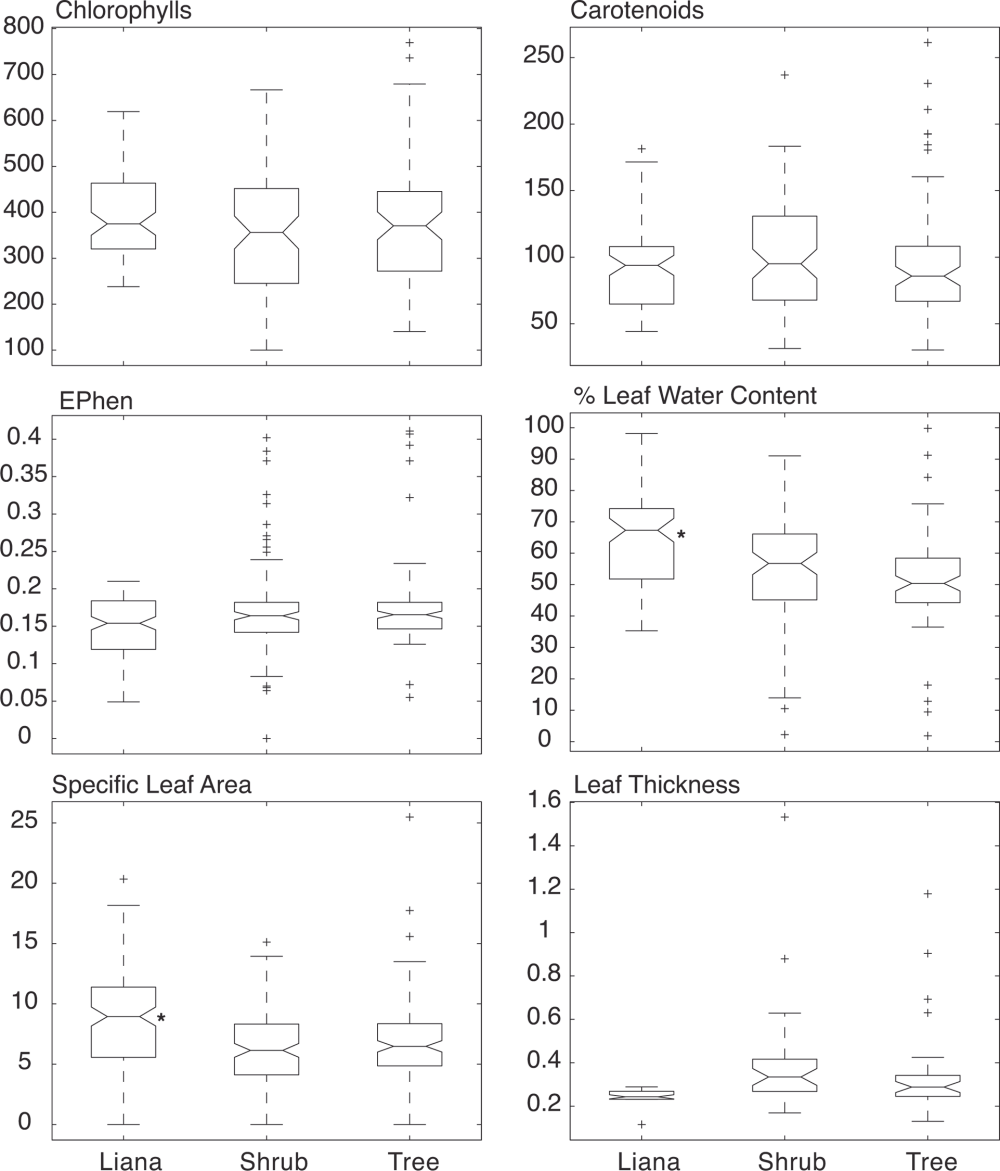
Comparison of leaf trait distributions between vegetation life forms by one-way ANOVAs. Means that significantly differ (p<0.01) are denoted with an asterisk. CHL = Total Chlorophylls A + B (mol), Car = Carotenoids (mol), EPhen = Polyphenol content,%H2O = Percentage. Leaf Water Conent, SLA = Specific Leaf Area (m2 kg-1), LeafThick = Leaf thickness (mm).

One-way ANOVAs on six traits revealed that pigments and polyphenols concentrations (Chlorophyll and carotenoids) were similar across trees, shrubs and lianas (Fig. 5). However, trees and shrubs significantly differed from lianas in terms of the percentage of leaf water content and SLA (Fig. 5). In lianas, water content and SLA averaged 65% and 9.03 m^2^ kg-1, while in shrubs and trees these values averaged 51% to 55% and 6.1 to 6.7, respectively (Table 1). Leaf thickness was also lower in lianas than in shrubs and trees, although not significantly different.

Finally, the spectral indices that were expected to respond to the categorization by functional groups did not show any significant correlation. Specifically, the water band index was poorly correlated to percentage water content (r^2^ = 0.03) observed between individuals. In addition, the patterns observed in water content versus water band index values did not show any clumping by life form (ANOVA, *p* = 0.93, *F* = 0.08).

A modified NDVI (mND_705_) developed by [32], performed much better in correlation with the observed chlorophyll (A and B) extractions than the three other indices designed for hyperspectral chlorophyll detection (Simple Ratio 704/774, Gitelson-Merzylak_A_ 750/700, and Gitelson-Merzylak_B_ 750/550) [33]. When the relationship of mND_705_ was compared between life forms, no significant difference was found (*p* > 0.05, Fig. 4).

## Discussion

The leaf-level pigment concentrations found in Cerrado woody plants were consistent with other studies of species in tropical deciduous ecosystems [15]. Fig. 3 shows the level of correlation between traits. SLA and dry weight showed a significant positive correlation with leaf thickness, but were negatively correlated to pigment concentrations. Interestingly, the correlation of leaf thickness to polyphenol concentration was strongly positive, suggesting that polyphenol concentrations increase with larger and thicker leaves. The negative correlation of Polyphenols to pigment concentration suggests there is less photo-protection in the presence of more pigments. Fig. 4 presents normal observed correlations between total Chlorophyll, Carotenoids, and SLA but without the possibility of distinguish between structural groups. [20] has reported similar results for lianas and trees from tropical rainforests in Panama.

Ecophysiological traits between shrubs and trees were not statistically different, presumably because of the overlap of species, which can also be observed in Fig. 4. In fact, the methodology adopted for this study distinguished shrubs and trees on the basis of plant height instead of relying on other aspects, such as the presence of multiple stems. Therefore, future long term work should address such question by tagging and monitoring such species until the fertile stage in which the correct life form is then assigned.

In relation to the first objective of this study, results suggest that life forms can be separated in terms of functional traits. Lianas, in particular, showed significant differences with respect to shrubs and trees in two functional traits: Percent leaf water content and Specific Leaf Area. These particular differences between life forms were also found by [20].

[20] reported higher values of SLA and water content in lianas than trees in dry forests of Central America. Their discussion supported the capture/conservation theory by [34] that indicated that lianas tended to have a higher rate of resource acquisition in contrast to trees that tend to focus more on the conservation of acquired resources. [35] also suggested that higher SLA is associated with less structural material relative to metabolic components, less internal shading and shorter gas diffusion paths, larger intercellular air spaces, and consequently greater carbon assimilation. Our results indicate that in the Cerrado, similarly to tropical dry forests, the lack of structural support in lianas represents an ecophysiological advantage over other life forms because it allows them to invest more in water acquisition and transport, which can help explain liana’s higher water content and larger intercellular air spaces [20].

The results of this study support the idea of using key leaf biochemical properties for investigating ecosystem functional diversity from remote sensing, specifically in the case of lianas, which differ in terms of growth and resource allocation strategies from the rest of woody plants. But results are constrained only to the plant spectra measured using the UNISPEC. Further work should involve a full range spectrometer (400–2500 nm) to observer more in-depth variability in the short wave infrared and even in the thermal infrared, where potential and significant differences could be observed. However, in regards to the second objective of the study, the variation among ecophysiological traits and their relationships to associated spectral indices was not consistent or strong across traits (see Fig. 4). Leaf water content was weakly correlated to the water band index values, which resulted as an artifact of the Unispec since significant noise increases close to 900 nm. Indices related to total chlorophyll varied in the strength and direction of their relationship, with the strongest positive relationship being with mND_705_. The former could have potential implications for remote sensing studies in Cerrado ecosystems but without a study that cover the full range of the spectral (400–2500 nm), conclusions can only be applied to the visible and near infrared region.

None of the indices were able to separate or discriminate between functional groups, which were driven for the narrow range of the UNISPECT. This inability is related to one of the main limitations of our study, which is the use of a limited range in the spectral wavelengths (400 nm—1050 nm). The use of this range decreased our ability to relate functional traits such as water content and SLA to spectra. This eventually may cause problems for remote sensing studies based on multispectral sensors, such as Landsat 8. [36] found that SLA, water content and all pigments had a high correspondence between predicted and actual (r^2^ > 0.7 for all) using wavelengths from 400 nm to 2500 nm. In fact, a substantial portion of spectral features corresponding to leaf traits occurred at wavelengths greater than 1000 nm, the range at which the UNISPEC sensor ends. This suggests that leaf reflectance considering the whole electromagnetic spectrum provides greater information to predict functional traits and that the range of the UNISPEC sensor, used in this study, does not capture the wavelengths that describe the structural arrangement within leaves that contribute significantly to leaf reflectance. The UNISPEC instrument is more suited to the analysis of plant signals that occur within its spectral range such as chlorophyll a and b, carotenoids, and anthocyanins [37].

## Conclusion

A significant amount of work is necessary in the Cerrado ecosystem to answer the elusive questions of “Are species spectrally different?; Can we detect this tree from space?; and Can we separate functional groups?”. It is clear that important technological and methodological advances have taken place in the last 10 to 15 years in the search for the identification and discrimination of species using hyperspectral techniques. Spectral variability, both between and within species, still remains high. Also, spectral response and its drivers are dependent of site conditions (e.g. micro-meteorology, soil moisture conditions) and phenology, which complicates the search for remote sensing solutions for species mapping at regional scales. As such, it seems more reasonable to focus first on the differentiation between structural groups, and specifically liana communities, which are increasing under global environmental change [40]. It is clear that work on the identifications of specific species tend to add a significant amount of error to regional classifications using hyperspectral airborne and space-borne sensors [43].

Our work indicates that the two strongest functional traits that drive plant differentiation in the Cerrado vegetation are Specific Leaf Area (SLA) and Water Content. SLA is related to leaf water use efficiency (e.g. [38–39]) and has varying effects relative to other whole-plant traits across the globe [40–41]. SLA appears to be a primary axis of variation between plant groups of the Cerrado, and is independent of other life history trade-offs within vegetation. Both SLA and water content are good candidates for spectral characterization. The ecological linkage to water balance and life strategies encourages these traits as starting points for modeling plant communities using hyperspectral remote sensing. Additionally, the affirmation of the importance of lianas as a biochemical and structurally distinctive group is a significant finding for future remote sensing applications directed towards the conservation of the Cerrado ecosystem. The apparent expansion of lianas in tropical forests in recent years suggests further changes in forest composition linked to changing rainfall patterns may continue to occur in tropical environments, with potentially large impacts on forest biodiversity [40–42].

To further explore the relationships between traits and observed spectra of plants, hyperspectral analysis considering the full electromagnetic spectrum are recommended. Shape comparisons [12], Spectral Angle Mapper (SMA), non-parametric classification methods, all use inclusive hyperspectral information to determine the relative variation within similar spectral responses. This can be used to discriminate among vegetation structural groups (e.g. lianas versus trees) or species. Unlike indices or feature detection, these types of remote sensing analysis are not focused on a specific region of the spectrum to test a specific variable but have class outcomes. This could be a very important first step in terms of community mapping rather than tackle the complex problem of species mapping on highly variable landscapes.

## References

[1] Myers N, Mittermeier RA, Mittermeier CG, da Fonseca GAB, Kent J (2000) Biodiversity hotspots for conservation priorities. Nature 403: 853–858. 10706275 10.1038/35002501

[2] Oliveira-Filho AT, Ratter JA (2002) Vegetation physiognomies and woody flora of the cerrado biome. In: Oliveira PS, Marquis RJ, editors. The Cerrados of Brazil. New York: Columbia University Press pp. 91–120.

[3] Holdridge LR (1967) Life Zone Ecology. San Jose, Costa Rica: Tropical Science Center 149 p.

[4] Ratter JA, Bridgewater S, Ribeiro JF (2006) Biodiversity patterns of the woody vegetation of the Brazilian Cerrado. In: Pennington T, Ratter JA, editors. Neotropical Savannas and Seasonally Dry Forests: Plant Diversity. Boca Raton: CRC Press pp. 31–66.

[5] Carvalho F, Souza FA, Carrenho R, Moreira FMS, Fernandes GW, et al (2012) The mosaic of habitats in the high-altitude Brazilian rupestrian fields is a hotspot for arbuscular mycorrhizal fungi. Appl Soil Ecol 52: 9–19.

[6] Ratter JA, Ribeiro JF, Bridgewater S (1997) The Brazilian Cerrado vegetation and threats to its biodiversity. Ann Bot 80: 223–230.

[7] Klink CA, Machado RB (2005) Conservation of the Brazilian Cerrado. Conserv Biol 19: 707–713.

[8] Diaz S, Cabido M (2001) Vive La Difference: Plant functional diversity matters to ecosystem processes. Trends Ecol Evol 16: 646–655.

[9] Turner W, Spector S, Gardiner N, Fladeland M, Sterling E, et al (2003) Remote sensing for biodiversity science and conservation. Trends Ecol Evol 18: 306–314.

[10] Gillespie TW, Foody GM, Rocchini D, Giorgi AP, Saatchi S (2008) Measuring and modelling biodiversity from space. Prog Phys Geogr 32: 203–221.

[11] Nagendra H (2001) Using remote sensing to assess biodiversity. Int J Remote Sens 22: 2377–2400.

[12] Price JC (1994) How unique are spectral signatures? Remote Sens Environ 49: 181–186.

[13] Tucker CJ (1979) Red and photographic infrared linear combinations for monitoring vegetation. Remote Sens Environ 8: 127–150.

[14] Tucker CJ (1980) Remote-sensing of leaf water-content in the near-infrared. Remote Sens Environ 10: 23–32.

[15] Kalacska M, Bohman S, Sanchez-Azofeifa GA, Castro-Esau K, Caelli T (2007a) Hyperspectral discrimination of tropical dry forest lianas and trees: comparative data reduction approaches at the leaf and canopy levels. Remote Sens Environ 109: 406–415.

[16] Alvarez-Añorve M, Quesada M, de la Barrera E (2008) Remote sensing and plant functional groups. In: Kalacska M, Sánchez-Azofeifa GA, editors. Hyperspectral Remote Sensing of Tropical and Sub-Tropical Forests. Boca Raton: CRC Press pp. 27–43.

[17] Castro-Esau KL, Sanchez-Azofeifa GA, Rivard B, Wright SJ, Quesada M (2006b) Variability in leaf optical properties of mesoamerican trees and the potential for species classification. Am J Bot 93: 517–530. doi: 10.3732/ajb.93.4.517 21646212 10.3732/ajb.93.4.517

[18] Sims DA, Gamon JA (2003) Estimation of vegetation water content and photosynthetic tissue area from spectral reflectance: a comparison of indices based on liquid water and chlorophyll absorption features. Remote Sens Environ 84: 526–537.

[19] Castro-Esau KL, Sanchez-Azofeifa GA, Caelli T (2004) Discrimination of lianas and trees with leaf-level hyperspectral data. Remote Sens Environ 90: 353–372.

[20] Sanchez-Azofeifa GA, Castro K, Joseph Wright S, Gamon J, Kalacska M, et al (2009) Differences in leaf traits, leaf internal structure, and spectral reflectance between two communities of lianas and trees: implications for remote sensing in tropical environments. Remote Sens Environ 113: 2076–2088.

[21] Alvarez-Añorve MY, Quesada M, Sánchez-Azofeifa GA, Avila-Cabadilla L, Gamon J (2012). Functional change, functional groups and spectroscopy in a highly diverse dry tropical system: an integrative evaluation of tropical dry forest succession and its practical implications. Am J Bot 99: 816–826. doi: 10.3732/ajb.1100200 22523349 10.3732/ajb.1100200

[22] Miura T, Huete A, Ferreira L, Sano E (2003) Discrimination and biophysical characterization of Cerrado physiognomies with EO-1 Hyperspectral Hyperion. Anais XI SBSR, Belo Horizonte, Brasil, 05–10 abril 2003, INPE, pp 1077–1082.

[23] De Souza AA, Galvao LS, Dos Santos JR (2001) Índices de vegetação derivados do sensor Hyperion/EO-1 para estimativa de parâmetros biofísicos de fitofisionomias de Cerrado. Anais XIV Simpósio Brasileiro de Sensoriamento Remoto, Natal, Brasil, 25–30 abril 2009, INPE, pp. 3095–3102.

[24] Cornelissen JHC, Lavorel S, Garnier E, Diaz S, Buchmann N, et al (2003) A Handbook of protocols for standardized and easy measurement of plant functional traits worldwide. Australian J Bot 51: 335–380.

[25] Terashima I, Hikosaka K (1995) Comparative ecophysiology of leaf and canopy photosynthesis. Plant Cell Environ 18: 1111–1128.

[26] Castro-Esau KL, Sanchez-Azofeifa GA, Rivard B (2006a) Comparison of spectral indices obtained using multiple spectroradiometers. Remote Sens Environ 103: 276–288.

[27] Goulas Y, Cerovic ZG, Cartelat A, Moya I (2004) Dualex: A New Instrument for Field Measurements of Epidermal Ultraviolet Absorbance by Chlorophyll Fluorescence. Appl Opt 43: 4488–4496. 15382317 10.1364/ao.43.004488

[28] Meyer S, Cerovic ZG, Goulas Y, Montpied P, Demotes-Mainard S, et al (2006) Relationships between optically assessed polyphenols and chlorophyll contents, and leaf mass per area ratio in woody plants: a signature of the carbon-nitrogen balance within leaves? Plant Cell Environ 29: 1338–1348. 17080955 10.1111/j.1365-3040.2006.01514.x

[29] Cartelat A, Cerovic ZG, Goulas Y, Meyer S, Lelarge C, et al (2005) Optically assessed contents of leaf polyphenolics and chlorophyll as indicators of nitrogen deficiency in wheat (Triticum Aestivum L.). Field Crops Res 91: 35–49.

[30] Wellburn AR (1994) The spectral determination of chlorophyll-a and chlorophhyll-b, as well as total carotenoids, using various solvents with spectrophotometers of different resolution. J Plant Physiol 144: 307–313.

[31] Holden M (1976) Chlorophylls. In: Goodwin TW, editor. Chemistry and Biochemistry of Plant Pigments. London: Academic Press pp. 1–37.

[32] Sims DA, Gamon JA (2002) Relationships between leaf pigment content and spectral reflectance across a wide range of species, leaf structures and developmental stages. Remote Sens Environ 81: 337–354.

[33] Gitelson AA, Merzlyak MN (1997) Remote estimation of chlorophyll content in higher plant leaves. Int J Remote Sens 18: 2691–2697.

[34] Pooter H, De Jong R (1999). A comparison of specific leaf area, chemical composition and leaf construction costs of field plans from 15 habitats differing in productivity. New Phytol 143: 163−176.

[35] Wright IJ, Reich PB, Westoby M (2001). Strategy-shifts in leaf physiology, structure and nutrient content between species of high and low rainfall, and high and low nutrient habitats. Funct Ecol 15: 423−434

[36] Asner GP, Martin RE (2009) Airborne spectranomics: mapping canopy chemical and taxonomic diversity in tropical forests. Front Ecol Environ 7: 269–276.

[37] Blackburn GA (2007) Hyperspectral remote sensing of plant pigments. J Exp Bot 58: 855–867. 16990372 10.1093/jxb/erl123

[38] Hoffmann WA, Franco AC, Moreira MZ, Haridasan M (2005) Specific leaf area explains differences in leaf traits between congeneric savanna and forest trees. Func Ecol 19: 932–940.

[39] Bucci SJ, Scholz FG, Goldstein G, Meinzer FC, Franco AC, et al (2006) Nutrient availability constrains the hydraulic architecture and water relations of savannah trees. Plant Cell Environ 29: 2153–2167. 17081249 10.1111/j.1365-3040.2006.01591.x

[40] Wright IJ, Reich PB, Westoby M, Ackerly DD, Baruch Z, et al (2004) The worldwide leaf economics spectrum. Nature 428: 821–827. 15103368 10.1038/nature02403

[41] Wright IJ, Ackerly DD, Bongers F, Harms KE, Ibarra-Manriquez G, et al (2007) Relationships among ecologically important dimensions of plant trait variation in seven neotropical forests. Ann Bot 99: 1003–1015. 16595553 10.1093/aob/mcl066 PMC2802905

[42] Phillips OL, Martinez RV, Arroyo L, Baker TR, Killeen T, et al (2002). Increasing dominance of large lianas in Amazonian forests. Nature 418: 770−774. 12181565 10.1038/nature00926

[43] Zhang J, Rivard B, Sanchez-Azofeifa GA, Castro-Esau K (2006). Intra and inter-class spectral variability of tropical tree species at La Selva, Costa Rica: Implications for species identification using HYDICE imagery. Remote Sens Environ 105: 129–141.

